# Associations between Depressive Symptoms and 30-day Hospital Readmission among Older Adults

**DOI:** 10.4172/2167-1044.1000185

**Published:** 2015-04-24

**Authors:** Ivonne M. Berges, Sania Amr, Danielle S. Abraham, Dawn L. Cannon, Glenn V. Ostir

**Affiliations:** Department of Epidemiology and Public Health, Division of Gerontology, University of Maryland School of Medicine, USA

**Keywords:** Depression, Older adults, Hospital readmission

## Abstract

**Background:**

Hospital readmissions are common and costly. Our goal was to determine the association between depressive symptoms and readmission within 30 days following hospital discharge in older adults.

**Methods:**

We analyzed data from a study of 789 persons aged 65 years or older admitted to a 20-bed acute care for elders (ACE) hospital unit from May 2009 to July 2011. Depressive symptoms were recorded within 24-hours of admission to the hospital unit, using the Center for Epidemiologic Studies -Depression (CES-D) Scale. The primary outcome was readmission to hospital within 30 days of discharge.

**Results:**

The mean age was 77 years; 66% were female, 72% were White, and 59% were unmarried. On average, older patients reported 2.6 comorbid conditions. Sixteen percent were classified with high depressive symptoms (CES-D ≥ 16). The readmission rate within 30 days was 15%. Older patients with high depressive symptoms had more than 1.6 times the odds (OR 1.66; 95% CI: 1.01-2.74) of being readmitted within 30-days, as compared to those with low depressive symptoms (CES-D < 16), after adjustment for age, race/ethnicity, sex, marital status and comorbid conditions.

**Conclusion:**

High depressive symptoms increased the risk of hospital readmission within 30 days of discharge after adjusting for relevant covariates. In-hospital screening for depressive symptoms may identify older persons at risk for recurrent hospital admissions.

## Introduction

Hospital readmission has received attention as a health care quality indicator and a factor that can reduce Medicare costs [1, 2]. The Patient Protection and Affordable Care Act (111th Congress, 2010) created a “hospital readmission reduction program” intended to assist hospitals with patient transitions from acute care and reward hospitals that are successful in reducing avoidable readmissions [3]. Research suggests that approximately twenty percent of Medicare hospitalizations are preventable readmissions occurring within thirty days following acute care discharge [4]. The annual cost of re-hospitalizations for Medicare patients is estimated at over $15 billion dollars with the majority of these readmissions considered preventable [5]. Although a large number of risk factors for early readmission has been documented in the literature [6, 7]; little attention has been given to the psychosocial factors of the patient including depression.

Depressive symptoms are common in older persons and symptoms of depression are known to increase during hospitalization [8]. After heart disease, it is projected that depression will become the second leading cause of disease burden by the year 2020 [9]. A World Health Organization survey study found depression produced the greatest declines in health status when compared to other chronic diseases including angina, arthritis, asthma, and diabetes [10]; yet depressive symptoms as a risk factor for early hospital readmission has been overlooked. Information on associations between depression at time of hospitalization and 30-day readmission can provide insight about vulnerable older patients and could potentially be used to predict preventable readmissions.

The objective of the study was to determine the association between depressive symptoms and 30-day readmission for older persons admitted to an acute care for elders (ACE) hospital unit. We hypothesized that older patients with high versus low depressive symptoms would be at significantly greater risk of hospital readmission within 30-days of discharge.

## Methods

The study population was drawn from a 20-bed ACE hospital unit at the University of Texas Medical Branch from May 2009 to July 2011. Patients included in the study were aged 65 years or older. To increase the generalizability of findings, the included sample had to have a primary admitting diagnosis of cardiopulmonary disease or respiratory problems or gastrointestinal problems. Together the three diagnoses account for 87% of admitting complaints to ACE hospital units in the US [11]. Patients were excluded from the study if at time of admission they were disorientated to person, place, or time per nursing assessment (18.4%), admitted for observation (< 24 hours) (14.0%), or transferred from a nursing home, intensive care unit, or day surgery settings (14.8%). Excluded patients did not differ significantly from those included with respect to age, sex or race/ ethnicity. The final sample included 789 older patients. The study received approval from the University’s institutional review board, and all patients provided written informed consent.

### Data collection

A trained clinical interviewer completed face-to-face interviews with patients within 36 hours of admission; chart abstractions, via the electronic medical record, were completed within 72 hours of admission. Information obtained included age in years (65–103), sex, race/ethnicity (non-Hispanic white, non-Hispanic black, or Hispanic), and number of comorbid conditions using the Charlson Comorbidity Index (based on the weighted score assigned to each comorbid condition with which a risk of dying is associated) [12, 13].

### Depressive symptoms

The 20-item Center for Epidemiologic Studies-Depression (CES-D) scale [13] was used to determine depression status at admission to the ACE unit. Responses were scored on a four-point scale (0–3) and ranged from 0 (rarely or none of the time) to (most of the time). Summing the responses created a summary score ranging from 0 to 60, with higher scores indicating more depressive symptoms. Summary scores were used to create a 4- (0-7, 8-1, 16-2, and ≥24) and 2-level (0-15 and ≥16) categorical measure [1, 14]. The reliability and validity of the CES-D scale has been established [11, 15, 16].

### Outcome

The primary outcome, any hospital readmission within 30 days following discharge, was documented by contacting the patient, or other individuals the patient designated as a contact, by telephone. Patient-reported hospital readmission was verified by examining hospital records. When a patient or family member could not be contacted, hospital records were reviewed.

### Statistical analysis

Sociodemographic and clinical characteristics were examined using descriptive statistics for continuous variables and contingency tables for categorical variables, with significance tests by t-test and χ2 analysis, respectively. Logistic regression [17] models were used to assess associations between depressive symptoms and readmission within 30 days of discharge, before and after adjustment for relevant covariates. The strength of the association was estimated using odds ratios (OR) and associated 95% confidence intervals (CI). Testing was 2-sided and p < 0.05 was considered significant. All analyses were performed using commercially available software (SAS statistical software, version 9., SAS Institute, Inc., Cary, NC.).

## Results

The sample included 789 older patients. Of these, 42% were aged 65-7, 44% were 75-8, and 14% were 85 and older, with an overall mean age of 76.9 (SD=6.53). Most were female (66%); and the majority were white (72%). Less than half (41%) were currently married. The average number of comorbidities was 2.6 (SD=2.06), with 48% of patients reporting three or more comorbidities. Sixteen percent reported high depressive symptoms (CES-D ≥ 16); and 15% of patients were readmitted within 30-days of hospital discharge. Table 1 shows patient sociodemographic characteristics and number of comorbidities by depression status.

Patients with high depressive symptoms were significantly younger than those with low depressive symptoms (CES-D < 16) (p < 0.01), but the former group did not significantly differ from the latter with respect to sex, race/ethnicity, marital status and comorbidities.

**Figure 1:**
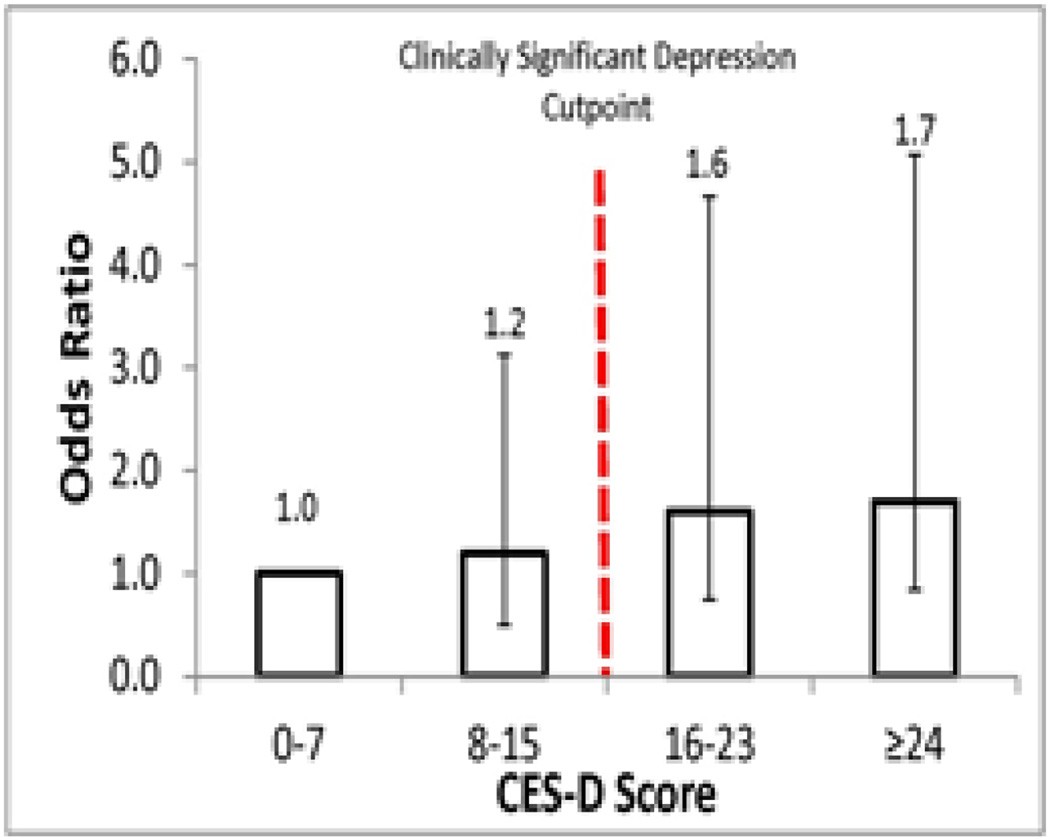
Odds ratios (and 95% confidence intervals) of being readmitted within 30-days of hospital discharge stratified across four CES-D categories.

Figure 1 shows the odds of 30-day readmission stratified by four categories of depressive symptom score (0-7, 8-1, 16-2, and > 24). One hundred and fifty-five (20%) and 508 (64%) patients were in the two lowest categories, respectively. Of those in the two highest depressive symptom categories, 69 (9%) had scores of 16-23 and 57 (7%) had scores ≥24. The figure suggests a possible threshold effect, with patients classified with high depressive symptoms (CES-D ≥ 16) showing an increased risk of 30-day readmission compared to those classified with low depressive symptoms (CES-D < 16). Of those with high depressive symptoms, 26 (21%) were readmitted to hospital within 30-days of discharge while 9 (14%) with low depressive symptoms (< 16) were readmitted within 30-days.

Table 2 shows the crude and adjusted association between depressive symptoms (CES-D < 16 and ≥ 16) and readmission. Although the association did not reach statistical significance, the crude model shows a strong increased risk of being readmitted among patients with high depressive symptoms (OR 1.60; 95% CI: 0.98, 2.59). After adjustment for age (continuous), sex, race, marital status, and comorbidities (continuous), the association between high depressive symptoms and readmission within 30 days following discharge was statistically significant (OR 1.66; 95% CI: 1.0, 2.74). Non-significant associations were found between the other covariates included in the model and 30-day readmission, with the exception of comorbidities (OR 1.20; 95% CI: 1.09, 1.31).

## Discussion

We examined depression status in hospitalized older persons and determined the risk of hospital readmission within 30 days of discharge. We found a significant association between high depressive symptoms and early hospital readmission after adjustment for covariates that included comorbid conditions. This finding suggests that scores of 16 or more on the CES-D scale may be an important threshold to stratify early readmission risk.

With a growing older population, a rise in the prevalence of depression is to be expected. Projections indicate that after heart disease, depression will become the second leading cause of disease burden by the year 2020 [10]. In a world health survey study, depression produced the greatest declines in health status when compared to other chronic diseases including angina, arthritis, asthma, and diabetes [9]. In our study we found that each comorbid illness was associated with an increased the odds of being readmitted by 20%, while high depressive symptoms were associated with a 66% increase.

Attention directed toward reducing 30-day hospital readmissions has increased substantially since the Patient Protection and Affordable Care Act (Public Law 111-148) was signed into law. Given the large number of risk factors for early readmission that have been documented in the literature [6, 7] and relatively poor performance of related prediction models [7] a single psychological health measure that captures the effects of disparate variables is potentially important. High depressive symptoms could provide physicians and other members of the health care team with critical information about the patient’s underlying mental health status and may capture unrecognized information about the potential severity or worsening of acute conditions that can result in early readmission. A WHO World Health survey suggests the need for timely screening and treatment of depressive symptoms [9

Mechanisms whereby depression influences health and leads to early readmission is likely complex and interactive [18–20]. Although a strong association between depression and chronic conditions clearly exists, whether this relationship ties directly to higher readmission risk, and subsequently poorer health outcomes is less clear. Our findings suggest that other mechanisms may play a role. For example, it is known that depressed individuals often do not follow medical recommendations for underlying medical illnesses and have lower treatment compliance [21–23]. Without proper attention to treatment, depression has the tendency to assume a chronic course, and over time to be associated with increasing disability [24, 25]. In turn, increased disability status reflects the integrated functioning of bodily systems including but not limited to the nervous, sensory, musculoskeletal and cardio-respiratory systems. With respect to hospital admission and readmission, addressing the exacerbation of disability due to depression needs to be more thoroughly investigated.

The current study has important strengths, including a representative sample of the three largest racial and ethnic groups in the U.S.: White, Black and Hispanic. Early readmission was ascertained by telephone and confirmed through chart review. The study also had limitations. Although our study design accounted for the three most common admitting diagnoses for ACE units, because this was a single site study lack of generalizability to other hospital units may be an issue. A second limitation was that depressive symptoms were assessed once during hospitalization. Information on depressive symptoms at other time points such as discharge may have provided valuable additional patient information on how change in depression status during hospitalization may signal future health needs. Finally, rather than a direct cause of early readmission, high depressive symptoms may be a marker of multiple factors affected by health and disease status and severity. Thus, research targeting pathways or mechanisms of action is needed to inform further work in this area.

In conclusion, our finding of a positive association between depression and readmission within 30 days of hospital discharge among older adults provide evidence to support the need for assessment of in-hospital depression status among this vulnerable population. Avoiding early and preventable hospital revisits is imperative to improving health care quality and reducing cost. The Medicare Payment Advisory Commission (MedPAC) has also made several recommendations related to monitoring and reducing hospital readmissions. As hospitals work to comply with these guidelines, the use of psychological screening tools as part of the standard hospital assessment would be a valuable means to identify older patients with the greatest risk for early readmission, and subsequently provide effective behavioral and educational interventions.

## Figures and Tables

**Table 1:** Sociodemographic and clinical characteristics by depression status (CES-D ≥ 16 vs. <16) among older adults admitted to acute care hospital unit.

Depression			
Variable	Yes (CES-D ≥ 16) (n=126)	No (CES-D <16) (n=663)	P-Value
Age, Mean (SD)	75. (6.0)	77. (6.5)	<0.01^†^
Sex, N (%)			0.99^*^
Male	(16.0)	226 (84.0)	
Female	8 (16.0)	437 (84.0)	
Race, N (%)			0.09^*^
Non-Hispanic White	98 (17.2)	47 (82.8)	
Non-Hispanic Black	1 (9.9)	128 (90.1)	
Hispanic	1 (18.2)	6 (81.8)	
Marital Status, N (%)			0.10^*^
Married	(13.3)	279 (86.7)	
Unmarried	8 (17.8)	38 (82.2)	
Comorbidities, Mean (SD)	2.9 (2.0)	2.6 (2.0)	0.07^†^

**Note:** test p-Value using Chi-Square^*^ or T-test^†^

**Table 2:** Crude and Adjusted Associations Between Depression (CESD scores ≥ 16) and Readmission within 30 days after Hospital Discharge Among Older Adults (n=789).

	Crude Model		Adjusted Model	
**Variable**	OR	95% CI	OR	95% CI
**Depression**				
**No (CES-D<16)**	1			
**Yes (CES- D ≥ 16)**	1.6	(0.98, 2.58)	1.66	(1.01, 2.74)
**Age (Continuous)**			1.02	(0.98, 1.05)
**Sex**				
**Female**			1	
**Male**			1	(0.65, 1.54)
**Race**				
**Non-Hispanic White**			1	
**Non-Hispanic Black**			1.42	(0.86, 2.34)
**Hispanic**			0.85	(0.41, 1.74)
**Marital Status**				
**Unmarried**			1	
**Married**			1.17	(0.76, 1.77)
**Comorbidities(Continuous)**			1.2	(1.09, 1.31)
